# The Effects of Secretion Factors from Umbilical Cord Derived Mesenchymal Stem Cells on Osteogenic Differentiation of Mesenchymal Stem Cells

**DOI:** 10.1371/journal.pone.0120593

**Published:** 2015-03-23

**Authors:** Kui-Xing Wang, Liang-Liang Xu, Yun-Feng Rui, Shuo Huang, Si-En Lin, Jiang-Hui Xiong, Ying-Hui Li, Wayne Yuk-Wai Lee, Gang Li

**Affiliations:** 1 Ministry of Education Key Laboratory for Regenerative Medicine, School of Biomedical Sciences, Faculty of Medicine, The Chinese University of Hong Kong, Hong Kong, China; 2 Stem Cells and Regenerative Medicine Laboratory, Li Ka Shing Institute of Health Sciences, Prince of Wales Hospital, The Chinese University of Hong Kong, Hong Kong, China; 3 Department of Orthopaedics & Traumatology, Faculty of Medicine, Prince of Wales Hospital, The Chinese University of Hong Kong, Hong Kong, China; 4 Lui Che Woo Institute of Innovative Medicine, Faculty of Medicine, The Chinese University of Hong Kong, Hong Kong, China; 5 Department of Orthopaedics, Zhongda Hospital, School of Medicine, Southeast University, Nanjing, China; 6 The CUHK-ACC Space Medicine Centre on Health Maintenance of Musculoskeletal System, The Chinese University of Hong Kong Shenzhen Research Institute, Shenzhen, China; 7 State Key Laboratory of Space Medical Fundamentation and Application, Astronaut Research and Training Center of China (ACC), 26 Beiqing Road, 100094, Beijing, China; Georgia Regents University, UNITED STATES

## Abstract

Factors synthesized by mesenchymal stem cells (MSCs) contain various growth factors, cytokines, exosomes and microRNAs, which may affect the differentiation abilities of MSCs. In the present study, we investigated the effects of secretion factors of human umbilical cord derived mesenchymal stem cells (hUCMSCs) on osteogenesis of human bone marrow derived MSCs (hBMSCs). The results showed that 20 μg/ml hUCMSCs secretion factors could initiate osteogenic differentiation of hBMSCs without osteogenic induction medium (OIM), and the amount of calcium deposit (stained by Alizarin Red) was significantly increased after the hUCMSCs secretion factors treatment. Real time quantitative reverse transcription-polymerase chain reaction (real time qRT-PCR) demonstrated that the expression of osteogenesis-related genes including ALP, BMP2, OCN, Osterix, Col1α and Runx2 were significantly up-regulated following hUCMSCs secretion factors treatment. In addition, we found that 10 μg hUCMSCs secretion factors together with 2×10^5^ hBMSCs in the HA/TCP scaffolds promoted ectopic bone formation in nude mice. Local application of 10 μg hUCMSCs secretion factors with 50 μl 2% hyaluronic acid hydrogel and 1×10^5^ rat bone marrow derived MSCs (rBMSCs) also significantly enhanced the bone repair of rat calvarial bone critical defect model at both 4 weeks and 8 weeks. Moreover, the group that received the hUCMSCs secretion factors treatment had more cartilage and bone regeneration in the defect areas than those in the control group. Taken together, these findings suggested that hUCMSCs secretion factors can initiate osteogenesis of bone marrow MSCs and promote bone repair. Our study indicates that hUCMSCs secretion factors may be potential sources for promoting bone regeneration.

## Introduction

Mesenchymal stem cells (MSCs) are multipotent progenitor cells derived from various tissues, such as bone marrow, hair follicles, muscle and umbilical cord. MSCs can differentiate into osteoblasts, adipocytes, chondrocytes, myoblasts and neurons [1, 2]. Especially, MSCs have outstanding osteogenic differentiation potential and they have been used to promote bone regeneration [3, 4].

Human umbilical cord derived mesenchymal stem cells (hUCMSCs) are a stem cell population that obtained from Wharton’s jelly, the main component of human umbilical cord matrix [5, 6]. hUCMSCs express adult stem cell markers including CD73, CD90, and CD105, as well as several embryonic stem cell properties [7]. hUCMSCs have a number of advantages over other cell sources in musculoskeletal tissue engineering. First, the use of postnatal hUCMSCs as the source of secretion factors is ethically uncomplicated. hUCMSCs are easy to isolate without requiring invasive procedures or generating ethical controversies. Also, the supply of hUCMSCs is abundant. hUCMSCs are obtainable in high numbers from *in vitro* culture. Umbilical cords are medical waste that can be collected at a low cost. Moreover, hUCMSCs appear to exhibit low immune rejection in animal studies [8, 9]. Furthermore, as a primitive MSCs population, hUCMSCs have higher multipotency compared with MSCs derived from other sources such as bone marrow or fat [5, 10–12]. In previous studies, hUCMSCs showed excellent potential for bone tissue engineering when cultured with three-dimensional (3D) scaffold both *in vitro* and *in vivo* [13–16].

MSCs synthesize a variety of bioactive factors including growth factors, cytokines, exosomes and microRNAs. Exosomes are nanometer-sized vesicles that are released into the extracellular matrices and outside the cells and function as mediators of intercellular communication. microRNAs and proteins may be transferred through exosomes [17–19]. MSCs are attracted to the damage sites where they produce secretion factors that enhance angiogenesis, reduce inflammation, promote tissue repair, and inhibit fibrosis and cell apoptosis [20–23]. It has been reported that hUCMSCs could produce various exosomes and trophic factors [24, 25]. Hsieh et al showed that umbilical cord derived MSCs induced better neural differentiation, neural cell migration and angiogenic properties compared with bone marrow derived MSCs through paracrine effect [26]. Moreover, the cytokines released by human umbilical cord blood derived MSCs could enhance osteoblast differentiation, cell proliferation and bone regeneration [23, 27]. However, the effects of secretion factors from hUCMSCs on osteogenic differentiation and bone regeneration has not been unveiled. Because both umbilical cord matrix and umbilical cord blood belong to umbilical cord tissue, the MSCs derived from them share numerous common properties. In addition, more cells could be harvested from umbilical cord matrix than that from umbilical cord blood. Thus we supposed umbilical cord matrix could be served as an ideal source for secretion factors that used in osteoblast differentiation and bone regeneration. The present study revealed the effects of hUCMSCs secretion factors on osteogenesis and the potential of using hUCMSCs secretion factors in the treatment of bone diseases.

Using hUCMSCs secretion factors in therapeutic approach has several advantages compared with using MSCs. hUCMSCs secretion factors could be applied directly to the damaged bone tissue in controlled dosage, space and time. Several limitations of cell therapy could be avoided when secretion factors of MSCs were employed, such as immune incompatibility, tumorigenicity and waiting time for *in vitro* expansion of cell preparation [28, 29].

In this study, we hypothesize that secretion factors from hUCMSCs could promote osteogenic differentiation and bone regeneration. First, we aim to demonstrate that hUCMSCs secretion factors could augment osteogenic differentiation of MSCs *in vitro*. We suppose the secretion factors from hUCMSCs contribute to accelerating bone regeneration, thus they possess the ability to induce osteogenic differentiation of MSCs. Then we intend to verify that secretion factors could enhance ectopic bone formation and calvarial bone defect repair *in vivo*. The hUCMSCs secretion factors could be applied conveniently to nude mice mode and rat mode as a pre-made product. As an alternative to cell therapy, the secretion factors from hUCMSCs may have therapeutic potential in treatment of orthopaedics diseases.

## Materials and Methods

### Ethics

Human fetal bone marrow mesenchymal stem cells were donated by the Stem Cell Bank of the Prince of Wales Hospital of the Chinese University of Hong Kong. Human ethics approval was obtained from the Joint CUHK-NTEC Clinical Research Ethics Committee of the Chinese University of Hong Kong (Reference No. CRE-2011.383) for the use of fetal human tissues. The informed written consent form was approved by the Clinical Research Ethics Committee and signed by the donor before sample collection.

For animal study, 10-week-old male nude mice and 20-week-old male Sprague-Dawley (SD) rats provided by animal house of the Chinese University of Hong Kong were used. Animal surgery was carried out under the animal license issued by the Hong Kong SAR Government and the approval of the Animal Experimentation Ethics Committee of the Chinese University of Hong Kong. All surgeries were performed under anesthesia, and efforts were made to minimize the suffering of the animals.

### Chemicals

All the chemicals used were purchased from Sigma USA, except where specified.

### Cell Culture and Phenotypes of MSCs

Human bone marrow derived mesenchymal stem cells (hBMSCs), hUCMSCs and rat bone marrow derived mesenchymal stem cells (rBMSCs) derived from Green fluorescent protein (GFP) transgenic rats were seeded in T75 flasks at a density of 1×10^3^cells/cm^2^ and cultured in Modified Eagle’s Medium of Alpha (α-MEM) (Invitrogen, USA) supplemented with 10% fetal bovine serum (FBS) (Gibco, USA) and 1% penicillin/streptomycin (Gibco, USA). The culture medium was changed every three days and the MSCs were kept under 5% CO_2_ at 37°C in a humidified condition. The cellular surface markers of MSCs were analyzed by flow cytometry. Briefly, cells at passage 3 or 4 were harvested and cell suspensions containing 1×10^5^ cells were incubated with 1 μl fluorescence conjugated antibodies: CD34, CD44, CD45, CD73, CD90, CD105, and corresponding isotype controls (all antibodies were purchased from BD Biosciences, USA) for 1 hour at 4°C. After washing with phosphate-buffered saline (PBS), the stained cells were resuspended in 500 μl stain buffer (BD Biosciences, USA) and immediately subjected to flow cytometric analysis using LSRFortessa Cell Analyzer (BD Biosciences, USA).

### The Preparation of Stem Cell Secretion Factors

With written informed consent, umbilical cords were obtained from patients after birth. Before the removal of blood vessels, the cords were treated with collagenase type I solution containing 300 U/mL collagenase type I (Sigma, USA), 1 mg/mL hyaluronidase (MP Biomedical, USA) and 3 mM calcium chloride (Fisher, USA), for 30 min at 37°C. Then the cords were minced and plated in a modified Dulbecco’s modified Eagle’s medium (DMEM) for 7 days as described previously[16]. The tissue remnants were then removed and the attached cells were harvested, washed, seeded at a density of 7500 cells/cm^2^ in T75 flasks and cultured in DMEM supplemented with 10% FBS and 1% antibiotics. The medium was replaced twice per week and cells were detached by 2.5% trypsin (Invitrogen, USA) when reaching 80% confluence. The culture medium was then changed to a plain α-MEM medium that contained no FBS, penicillin/streptomycin, or phenol red. Next, 20 ml conditioned serum free medium was collected at day 3 (at least 1×10^6^ cells in each flask) and filtered with a 0.2 μm filter. hUCMSCs from each individual were inspected by flow cytometry to confirm the phenotypes before they were used for secretion factors preparation. The secretion factors of hUCMSCs derived from different individuals were blended together. The quality of secretion factors of different batch was controlled to minimize the variation. Finally, the solution was lyophilized and stored in powder form at -80°C. For *in vitro* experiments, 100 μg powder of hUCMSCs secretion factors was dissolved in 1ml PBS and then added as a supplement to the culture medium at a concentration of 20 μg/ml.

### MTT Assay

MSCs were placed on a 96-well plate at a density of 5000 cells/cm^2^. After incubation for 24 h, the medium was changed for α-MEM medium containing 20 μg/ml hUCMSCs secretion factors. Normal α-MEM medium served as control. Then cells were incubated at 37°C for 3 days or 5 days (n = 5). For the MTT assay, 10 μl MTT (5 mg/ml) was added to the medium and the cells were left for 4 hours in the incubator. Then the supernatant was replaced with 100 μl Dimethyl sulfoxide, the plate was covered with tinfoil and vibrated on an orbital shaker for 15 min. Absorbance was measured at 570 nm using a VICTOR3 VTM Multilabel Counter (Perkin Elmer, USA).

### Osteogenic Differentiation of Human MSCs

MSCs were trypsinized and placed in a 12-well plate or a 24-well plate at a density of 4000 cells/cm^2^. The cells were incubated in α-MEM medium for three or four days. When over 80% confluence was reached, the medium was then replaced by an osteogenic induction medium (OIM) containing 1 nM dexamethasone, 20 mM beta-glycerophosphate, and 50 μM ascorbic acid. MSCs were treated for two weeks. The osteogenic differentiation was evaluated by real time qRT-PCR after 11 days and Alizarin Red staining at day 3, day 5, day 7 and day 11. Triplicate tests were conducted in each experiment.

### Alizarin Red Staining

The formation of mineralized matrix nodules was determined by Alizarin Red staining. In brief, the monolayer cells were washed with PBS and fixed with 70% ethanol for 30 min, then the cells were washed with distilled water and stained with 2% Alizarin Red (pH 4.0) for 5 min at room temperature. The cells were rinsed five times using distilled water to reduce nonspecific Alizarin Red staining before photos were taken using a digital scanner (Epson Perfection V700 Photo, Epson America. Inc., USA). For quantification of the mineralization, the monolayer was eluted with 10% (w/v) cetylpyridinium chloride for 15 min at room temperature, and the absorbance was measured at 570 nm.

### RNA Extraction and Quantitative Real-Time PCR

Cellular mRNA was isolated using a PureLink RNA Mini Kit (Ambion, USA) according to the manufacturer’s instructions. First-strand cDNA was synthesized by M-MLV reverse transcriptase (Promega, USA). Quantitative PCR amplification was performed in 96-well plates using SYBR green PCR master mix (Applied Biosystems, USA) and ABI Prism 7700 sequence Detection System (Applied Biosystems, USA)The mRNA expression of early and late osteogenic markers including Alkaline phosphatase (ALP), Bone morphogenetic protein 2 (BMP2), Collagen type 1α (Col1α), Osteocalcin (OCN), Osteopontin (OPN), Osterix, Runt-related transcription factor 2 (Runx2) was examined using the primer sets outlined in Table 1. The amplification procedure was carried out for 40 cycles, first at 95°C and held for 10 min, and then at 95°C for 15 sec and 60°C for 1 min in each cycle. Quantitative analysis was performed according to the ABI protocol. The threshold cycle value was calculated from amplification plots. Relative quantification of gene expression was determined using the delta delta CT (ΔΔCT) method. Glyceraldehyde-3-phosphate dehydrogenase (GAPDH) was used as the internal control to evaluate the relative expression.

**Table 1:** Primer sequences for qRT-PCR.

Gene name	Forward primer sequence (5′- 3′)	Reverse primer sequence (5′- 3′)
GAPDH	GGCATGGACTGTGGTCATGAG	TGCACCACCAACTGTTAGC
ALP	ACCATTCCCACGTCTTCACATTT	AGACATTCTCTCGTTCACCGCC
BMP2	GTATCGCAGGCACTCAGGTC	CACTTCCACCACGAATCCAT
Col1α	CACTGGTGATGCTGGTCCTG	CGAGGTCACGGTCACGAAC
OCN	CCTCACACTCCTCGCCCTATT	CCCTCCTGCTTGGACACAAA
OPN	GTACCCTGATGCTACAGACG	TTCATAACTGTCCTTCCCAC
Osterix	CCAGGCAACACTCCTACTCC	GCCTTGCCATACACCTTGC
Runx2	ACTTCCTGTGCTCGGTGCT	GACGGTTATGGTCAAGGTGAA

### Ectopic Bone Formation Assay

Resorbable porous hydroxyapatite/tricalcium phosphate (HA/TCP) was used as scaffold. We had three groups with five replicates in each group: hUCMSCs secretion factors + PBS, hBMSCs + PBS, and hBMSCs + hUCMSCs secretion factors. 2×10^5^ hBMSCs were loaded on the sterilized scaffolds (2 mm in diameter, 2 mm in length) and incubated for 3 hours at 37°C for cell attachment. 50 μl hUCMSCs secretion factors (200 μg/ml) or PBS were loaded onto each scaffold before implantation. Five male nude mice (10 weeks, body weight 20–25g) were placed under general anesthesia followed by disinfection of the dorsal back. Two small incisions were made parallel to the spinal column. Subcutaneous pockets were created by blunt dissection on each flank. Scaffolds of different groups were placed subcutaneously in each mouse at the dorsal side. Grafts were distributed randomly over four different implant positions and then the wounds were stitched. Animals were sacrificed 8 weeks after implantation. Samples were harvested and processed for histological examination. The osteoid matrix areas were measured using ImageJ software, five microscopic fields were chosen from each sample and measured as reported previously [30].

### Rat Calvarial Bone Critical Defect Model

Eight SD male rats (20 weeks, body weight 250–320g) were used. Rat bone marrow MSCs from GFP transgenic rat was used as the allogenic cell source, and 2% hyaluronic acid hydrogels was used as scaffold. All animals were placed under general anesthesia with a dosage of 0.2ml/100g body weight via intraperitoneal injection of a combination of ketamine, xylazine, and saline at a ratio of 3:2:3. The dorsal part of animal’s cranium was shaved and disinfected with an iodine solution, then mounted onto a stereotaxic frame for stereotactic control. An approximately 15 mm midline incision extending the length of the skull was made in the sagittal plane across the cranium. The skin and underlying tissues including the periosteum were detached to expose the parietal bones on both sides. Two bilateral symmetric 5 mm full thickness circular bones were removed in each parietal region of the cranium using a hollow trephine bur with a 5 mm outer diameter. Continuous irrigation with sterile PBS was used to prevent overheating of the bone margins and to maintain moisture in the tissue. Also, special attention was paid to ensure at least 2 mm of bone was left between two defects. Any animal with evidence of meninges injury or continuous hemorrhaging was excluded. 50 μl of 2% hyaluronic acid hydrogel (5 mm-diameter cylinder) with 1×10^5^ rBMSCs and 10 μg hUCMSCs secretion factors was immediately implanted into the right defect cavity. As negative control, 2% hyaluronic acid hydrogel with 1×10^5^ rBMSCs and PBS was implanted into the left side. The periosteum and scalp were closed by suture. Animals were allowed to move following recovery from the anesthesia and were sacrificed by overdose of pentobarbital at 4 weeks (n = 4) and 8 weeks (n = 4) after surgery. The defect sites were removed, including sufficient parietal bone and soft connective tissues surrounding the defect areas.

### Micro-Computed Tomography Imaging Analysis

Micro-computed tomography (MicroCT) was used for quantitative evaluation of the bone formation. The samples were imaged using a high-resolution 70 kVp scan by microCT machine (VivaCT, Scanco Medical, Bassersdorf, Switzerland). The 3D reconstruction was performed using standardized segmentation parameters (sigma: 0.8, threshold: 220–1000), which were kept constant through the scan. Circular contour lines were drawn around the defect area (diameter = 5 mm) excluding the neighboring native bone. The 3D reconstructive images of samples were generated from 2D slices by machine built-in software. The bone volume within the selected circular defect was calculated using the quantitative 3D evaluation program included in the microCT software package.

### Histology and Immunochemistry Examinations

The bone samples (two defects remained in one piece of cranial bone, 15 mm×10 mm approximately) were fixed in 10% formalin-buffered solution at room temperature, then decalcified in 9% formic acid for 3 weeks at room temperature on a rotating rocker. After gradient dehydration in ethanol, the samples were embedded in paraffin and sectioned (thickness = 5 μm) in the transverse plane. Sections were subjected to hematoxylin and eosin (H&E) staining or Safranin O staining.

For immunochemistry staining, the sections were immersed in 10 mM citrate buffer at 60°C for 20 min for antigen retrieval. Then sections were blocked in 5% goat serum and 1% BSA for 30 min, then incubated with the mouse anti-osteocalcin (1:1000, Santa Cruz, USA) or rabbit anti-GFP (1:1000, Santa Cruz, USA) overnight at 4°C. After being washed with PBS for three times, sections were incubated with the goat anti-mouse antibody (1:1000, Santa Cruz, USA) or goat anti-rabbit antibody (1:1000, Santa Cruz, USA) at room temperature for 1 hour. Then, the signal was developed by incubating using the DAB detection system (Dako, USA) for 1 min and observed under a microscope. The osteoid matrix areas were measured using Image J software. Five microscopic fields were chosen randomly from each sample and measured.

### Statistical Analysis

Values were presented for each group as mean (SD). The independent t-test, one-way ANOVA or paired t-test was used for comparison of the mean values between different groups using SPSS (version 16.0; SPSS Inc., Chicago, IL, USA). p < 0.05 was regarded as statistically significant.

## Results

### Characterization of Surface Markers of MSCs

Passage 3 hBMSCs were analyzed for surface antigens expression using flow cytometry. The cells expressed mesenchymal stem cells markers including CD73, CD90 and CD105, and matrix marker, CD44, while hBMSCs were negative for hematopoietic markers, CD34 and CD45 (S1 Fig.). These cells presented the characterization of MSCs and they were used in the following experiments.

The hUCMSCs were isolated from umbilical cord matrix of a number of donors and inspected for the surface markers by flow cytometry analysis. The hUCMSCs at passage 3 or 4 were prominently (above 95%) positive for CD44, CD73, CD90 and CD105, while they were negative for CD34 and CD45.

### UCMSC Secretion Factors Had no Effect on MSCs Proliferation

**Fig 1:**
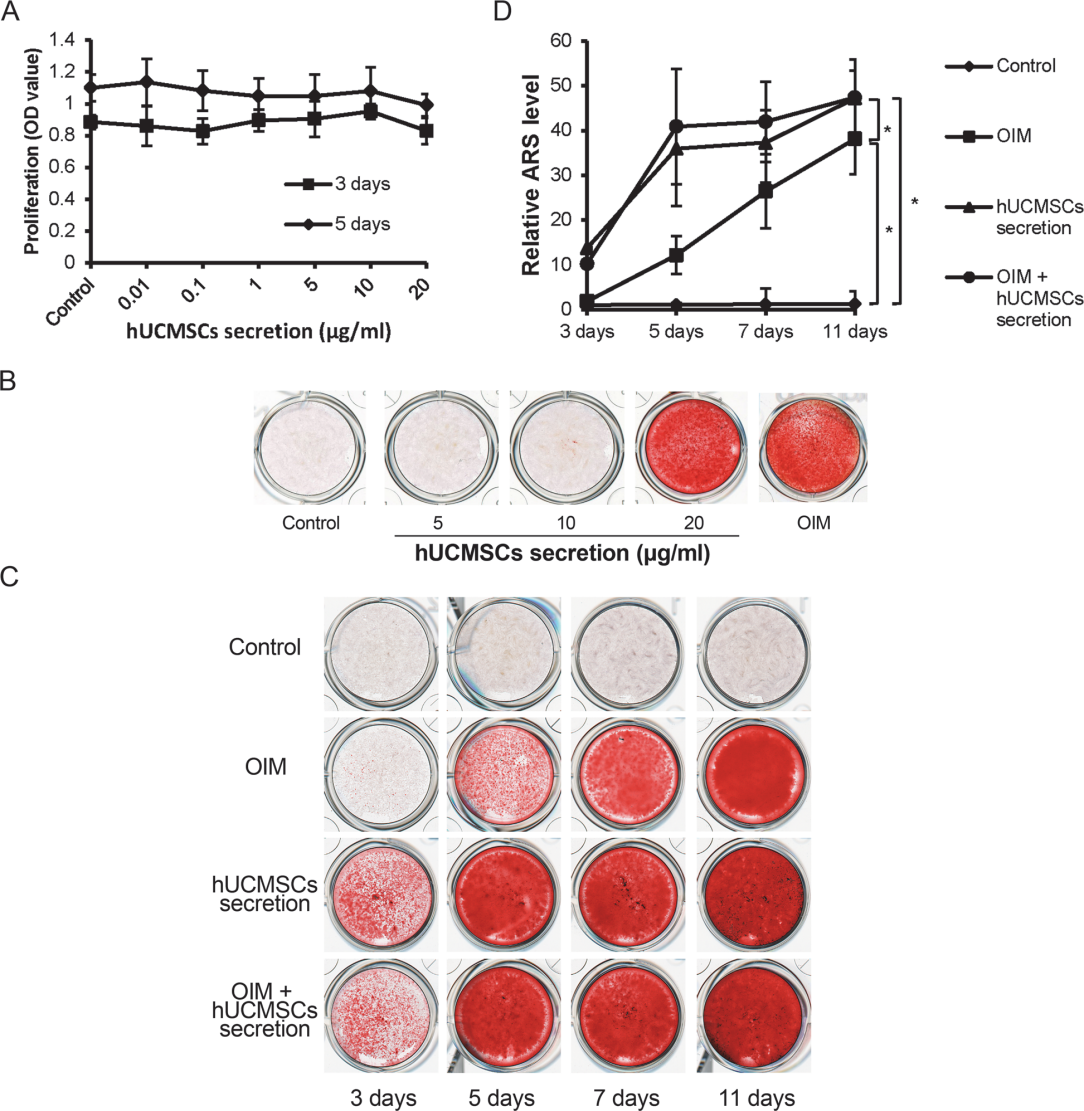
hUCMSCs secretion factors promote mineralization in osteogenic differentiation of hBMSCs. (A) hBMSCs were treated with hUCMSCs secretion factors (from 0.01 μg/ml to 20 μg/ml) for 3 days or 5 days. MTT assay was performed to test the cell proliferation rate. DMSO served as control. The hUCMSCs secretion factors have no significant effect on cell proliferation. (B) The dose-dependent assay of hUCMSCs secretion factors (5, 10 and 20 μg/ml) on osteogenic differentiation of hBMSCs that were treated for 11 days in 24-well plates. Calcium deposits formation was rare in the 5 μg/ml or 10 μg/ml group, whereas significant calcium nodules were formed in the 20 μg/ml group. (C) Time-dependent assay of hUCMSCs secretion factors at a dose of 20 μg/ml on osteogenic differentiation of hBMSCs that were treated for 3, 5, 7 and 11 days in 24-well plates. Alizarin Red staining showed that the amount of calcium deposits in both hUCMSCs secretion factors and OIM + hUCMSCs secretion factors groups were higher than those of the OIM group at all the time points. (D) Alizarin Red was dissolved in 10% (w/v) cetylpyridinium chloride, and the amount was determined by absorbance measurement at 570 nm. The amount of Alizarin Red in both hUCMSCs secretion factors and OIM + hUCMSCs secretion factors groups was significantly higher than that in the control group at day 3, 5, 7 and 11. (OIM: osteogenic induction medium. hUCMSCs secretion concentration: 20 μg/ml * p<0.05, compared to control).

The effect of hUCMSCs secretion factors on hBMSCs proliferation was examined by MTT assay. hUCMSCs secretion factors showed no effect on hBMSCs proliferation at concentrations of 0.01 μg/ml, 0.1 μg/ml, 1 μg/ml, 5 μg/ml, 10 μg/ml or 20 μg/ml (Fig. 1A). Based on these observations, hUCMSCs secretion factors was used at the dose range of 5 μg/ml to 20 μg/ml for the following evaluations on differentiation of MSCs.

### hUCMSCs Secretion Factors Promoted Osteogenic Differentiation of MSCs

**Fig 2:**
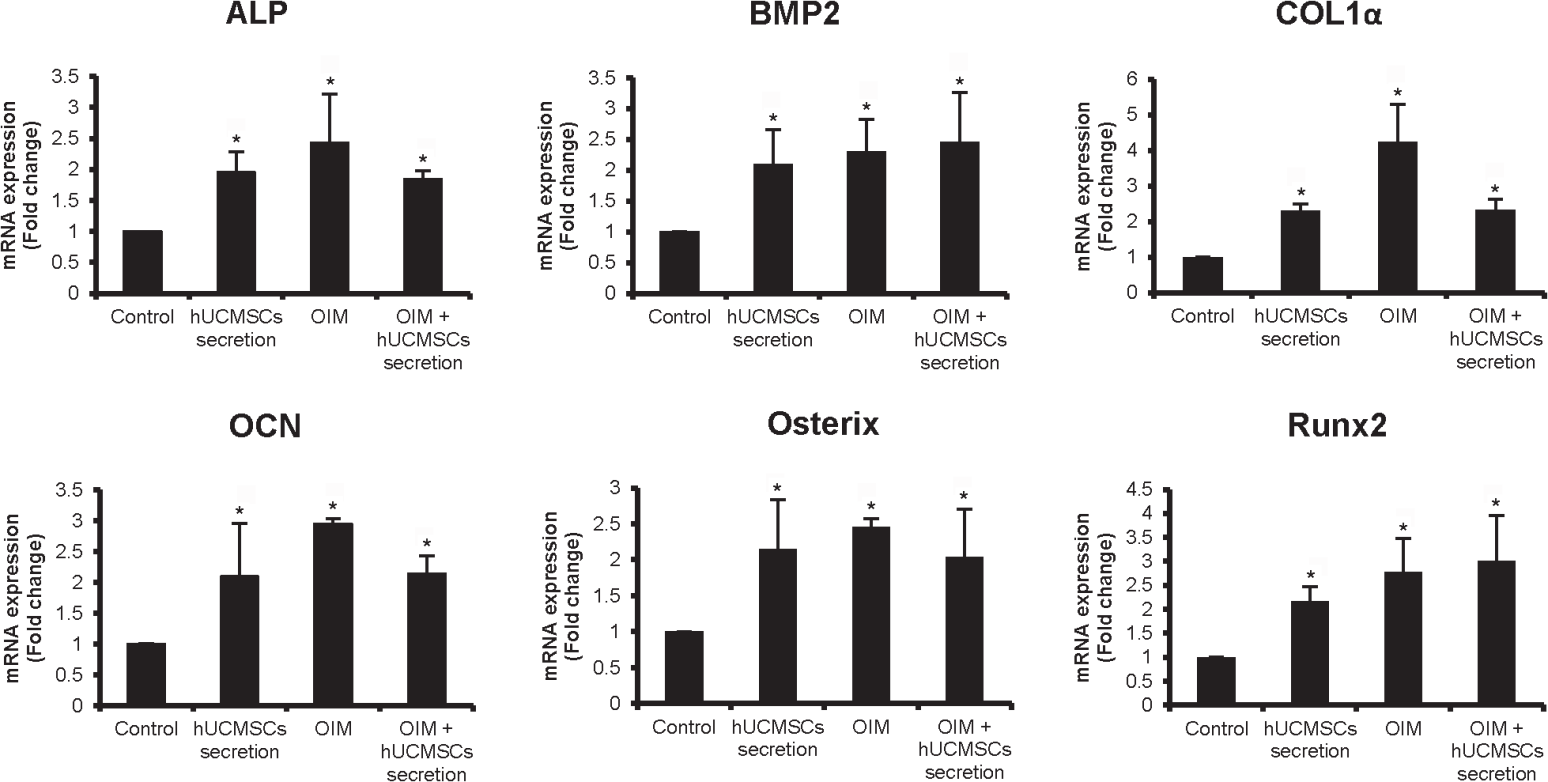
hUCMSCs secretion factors up-regulated expression of osteogenic marker genes of hBMSCs. hBMSCs were incubated with 20 μg/ml hUCMSCs secretion, osteogenic induction medium and osteogenic induction medium with 20 μg/ml hUCMSCs secretion for 11 days before RNA extraction and real time qRT-PCR. The expression of ALP, BMP2, Col1α, OCN, Osterix and Runx2 was significantly increased in the hUCMSCs secretion factors group, OIM group and OIM + hUCMSCs secretion factors group compared to that of the control group. Cells treated with α-MEM medium served as a control. (* p<0.05, compared to control)

In order to evaluate the optimal concentration of hUCMSCs secretion factors on osteogenic differentiation of MSCs, 5 μg/ml, 10 μg/ml and 20 μg/ml hUCMSCs secretion factors were tested. Alizarin Red staining showed that calcium nodule formation in the 5 μg/ml or 10 μg/ml group was rare, whereas numerous calcium deposits were seen in the 20 μg/ml group after 11 days treatment (Fig. 1B). Based on this observation, the dose 20 μg/ml was used as the optimal concentration in the following experiments. In a time-dependent assay, hBMSCs were treated with 20 μg/ml UCMSC secretion factors, OIM or OIM + 20 μg/ml hUCMSCs secretion factors respectively for 3 days, 5 days, 7 days or 11 days. AR staining showed that the calcium deposits in both hUCMSCs secretion factors and OIM + hUCMSCs secretion factors groups were more numerous than those of the OIM group at all the time points. (Fig. 1C). For quantification, the AR staining was dissolved in 10% (w/v) cetylpyridinium chloride and the absorbance was measured. The amount of Alizarin Red in both hUCMSCs secretion factors and OIM + hUCMSCs secretion factors groups was significantly higher than that in the control group at day 11. However, the difference between the hUCMSCs secretion factors groups with or without OIM was not obvious (Fig. 1D). In addition, the real time qRT-PCR results demonstrated an increase in the expression of ALP, BMP2, Col1α, OCN, Osterix and Runx2 in the hUCMSCs secretion factors and OIM + hUCMSCs secretion factors groups when compared to the control group at day 11 (Fig. 2).

### hUCMSCs Secretion Factors Together with MSCs Promoted Ectopic Bone Formation in Nude Mice

**Fig 3:**
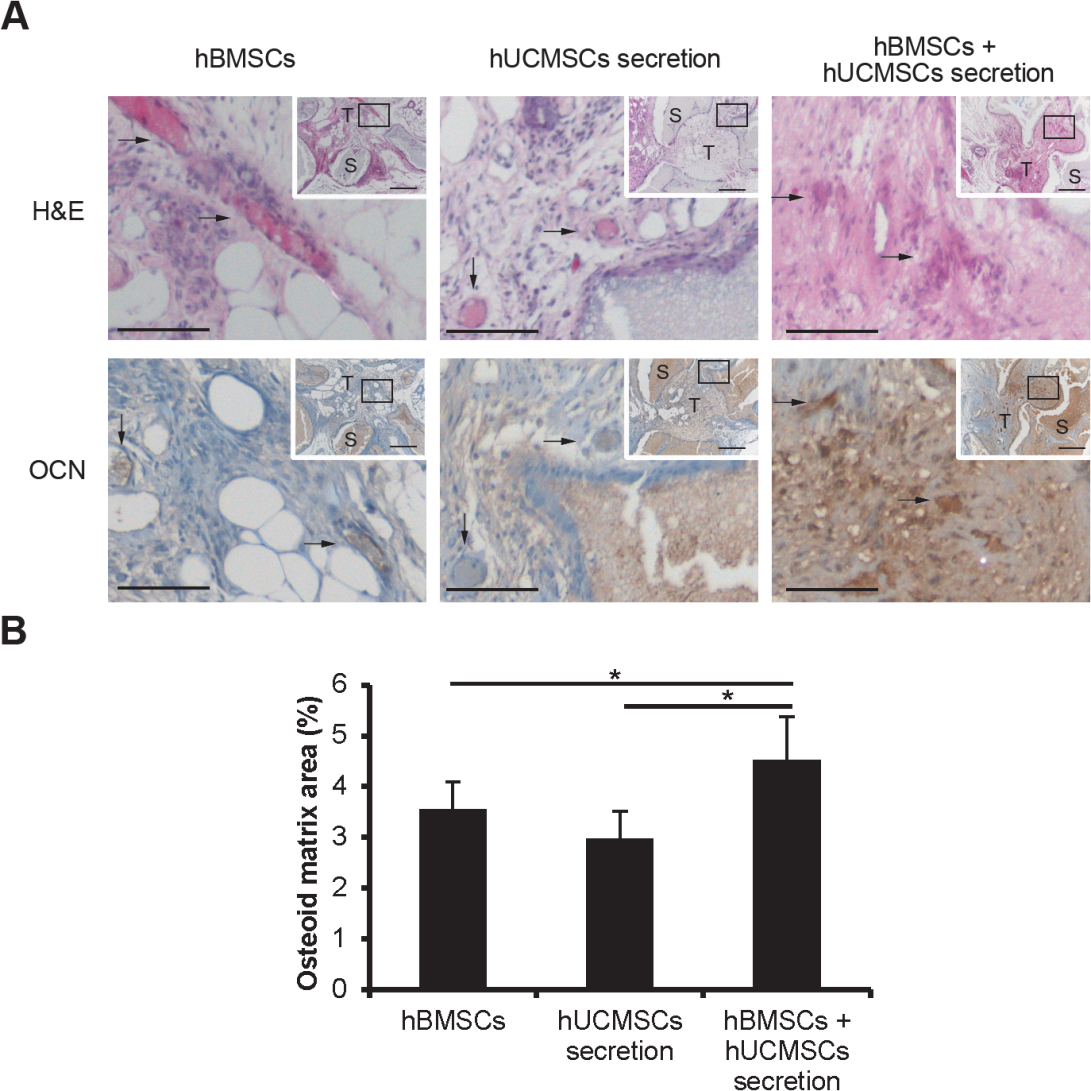
hUCMSCs secretion factors together with hBMSCs promoted ectopic bone formation in nude mice. (A) The sections were stained with H&E and OCN immunostaining. Arrows indicate the areas of osteoid matrices(H&E staining) or OCN positive cells (OCN immunostaining). OCN positive cells distributed in the areas of osteoid, which was consistent with that in H&E staining. The data showed that there was more OCN positive staining in the hBMSCs + hUCMSCs secretion factors group than in either of the other two groups. (B) Quantification of osteoid matrices areas showed significantly more osteoid matrices in the MSCs + hUCMSCs secretion factors group compared to the other two groups. Box areas of inset indicate the region shown in higher magnification. Scale bars = 100 μm (A), 200 μm (inset of A). S: HA/TCP scaffolds. T: regenerated tissue. *p<0.05.

To evaluate the effect of hUCMSCs secretion factors on ectopic bone formation, HA/TCP scaffolds were used in the nude mice implantation. We had three groups: hBMSCs (2×10^5^/graft), hUCMSCs secretion factors (10 μg/graft), and hBMSCs + hUCMSCs secretion factors (n = 5). Cells and secretion factors were loaded onto sterilized resorbable HA/TCP bone graft substitutes and implanted subcutaneously at the dorsal sides of nude mice. The osteoid matrices were formed by the hBMSCs implanted or autologous MSCs from surrounding tissues and limited subcutaneous blood flow. H&E staining was used to assess the formation of osteoid matrix after 8 weeks of transplantation. The sections were also subjected to immunohistochemical analysis to inspect the distribution of OCN. Results of H&E staining and immunohistochemistry showed new bone formation in the pores of grafts. Also, more bone tissue formation was observed in the hBMSCs + hUCMSCs secretion factors group than in either the hBMSCs or hUCMSCs secretion factors alone groups (Fig. 3A). The amount of osteoid matrix was increased in the hBMSCs + hUCMSCs secretion factors group compared to the hBMSCs and hUCMSCs secretion factors groups (Fig. 3B).

### hUCMSCs Secretion Factors Enhanced Rat Calvarial Bone Defect Repair

**Fig 4:**
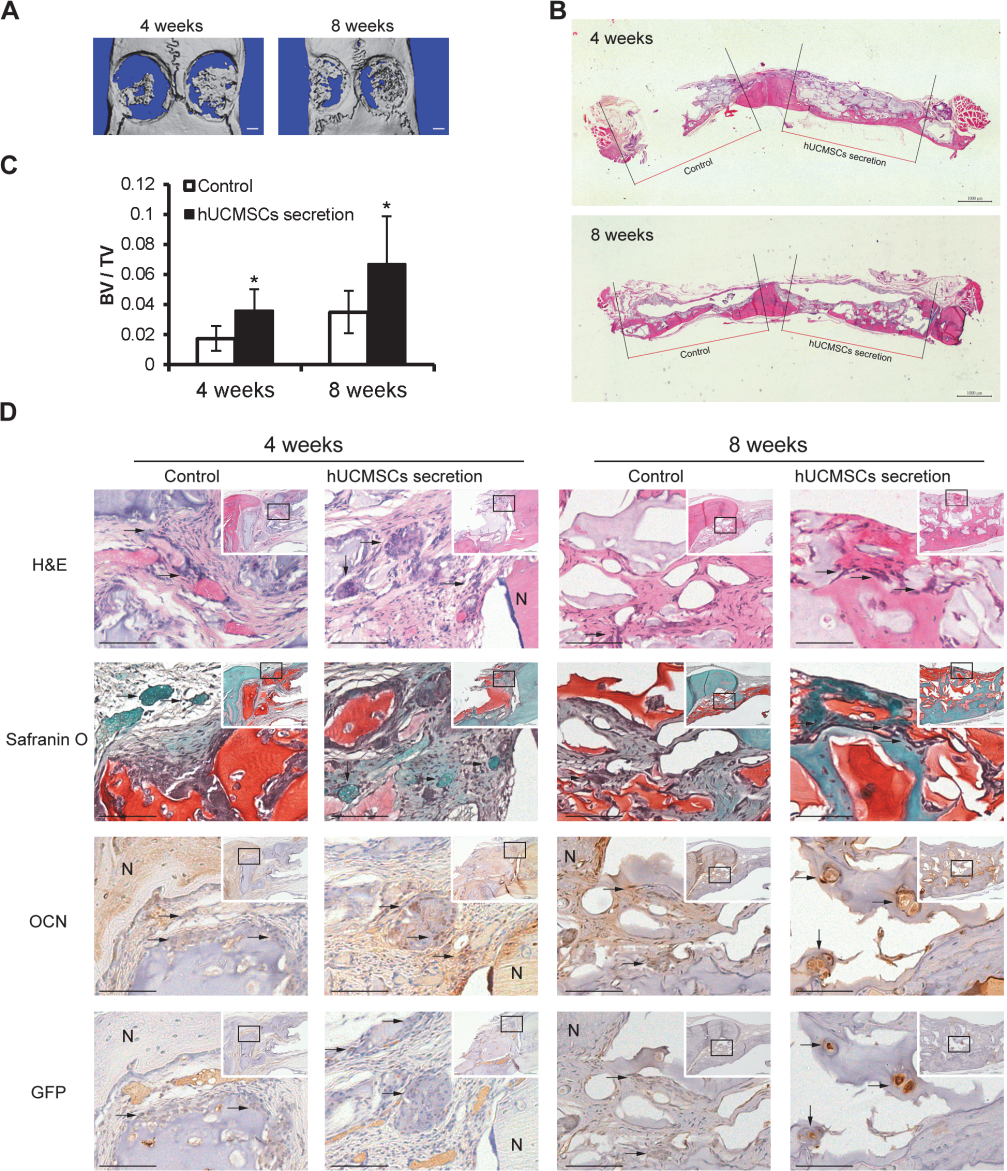
hUCMSCs secretion factors with rBMSCs enhanced rat calvarial bone repair. (A) 3D reconstruction of calvarial bone samples of 4 weeks and 8 weeks following treatment. The left side: PBS +GFP-rBMSCs; the right side: 10 μg hUCMSCs secretion + GFP-rBMSCs. The image showed increased bone formation in the hUCMSCs secretion factors group (the right side) than in the control group (the left side) at 4 weeks and 8 weeks. (B) H&E staining of cranial bone (transverse plane) showed increased regenerated tissue in hUCMSCs secretion factor group (the right side). (C) microCT analysis showed the new bone volume in the hUCMSCs secretion factors group was significantly increased compared to that of the control group at both time points. (D) The sections were stained with H&E, Safranin O, and immunostaining with OCN and GFP. OCN positive immunostaining illustrated the bone tissues. GFP positive cells were derived from allogenic rBMSCs in new tissue. More OCN and GFP positive cells were seen in the hUCMSCs secretion factors group than in the control group. Arrows indicate the newly formed bone tissues (in H&E and Safranin O staining), or positive cells (in OCN and GFP immunostaining). Box areas of inset indicate the region shown in higher magnification. Scale bars = 1000 μm (A,B), 100μm (D), 200 μm (inset of D). N: native bone. *p<0.05.

To investigate the effect of hUCMSCs secretion factors on bone repair, the rat calvarial bone critical defect model was used. microCT reconstruction and H&E staining showed that the new bone tissue appeared not only along the border but also in the center of the defect area. The bone repair was more enhanced in the hUCMSCs secretion factors group (right side) than it was in the control group (left side) at both 4 weeks and 8 weeks (Fig. 4A and B). The ratio of bone volume / total volume in the hUCMSCs secretion factors group was significantly increased compared to that of the control group at 4 weeks and 8 weeks (Fig. 4C). H&E staining results showed that new bone formed in the defect area next to the native bone. A large amount of cartilage formation was observed in the defect area as shown by safranin O staining. OCN immunostaining confirmed the bone tissues in the native bone and the defect area. A majority of GFP-positive cells were not presented in the native bone but only in the defect area, indicating that allogenic rBMSCs could be traced after transplanted. Further, GFP signals were detected in new bone and cartilage tissues, suggesting that allogenic rBMSCs contributed to the tissue regeneration in the defect area. More OCN and GFP positive cells appeared in the hUCMSCs secretion factors group than in that of the control group (Fig. 4D).

## Discussion

hUCMSCs are a population of MSCs derived from human umbilical cord matrix [5]. hUCMSCs secrete various factors including proteins, cytokines, chemokines and growth factors through paracrine effect [26, 31]. MSCs secretion factors derived from tissues other than umbilical cord matrix exhibit positive effect on osteogenesis and bone regeneration [23, 27].

In the present study, we investigated the effect of hUCMSCs secretion factors on osteogenic differentiation of hBMSCs and bone regeneration. The results demonstrate that hUCMSCs secretion factors could initiate osteogenic differentiation of MSCs and promote bone regeneration. In order to verify the effect of hUCMSCs secretion factors on MSC proliferation, a wide range of concentrations was examined. We found that hUCMSCs secretion did not affect the proliferation of MSC up to 20 μg/ml in the present *in vitro* study. A higher concentration was not tested due to economic concerns. The serum concentration of hUCMSCs secretion would be much lower if the same dose were used *in vivo*, indicating that hUCMSCs secretion would be safe for MSCs and without toxic effects for *in vivo* applications.

To investigate the effect of hUCMSCs secretion factors on osteogenic differentiation of MSCs, Alizarin Red staining and real time qRT-PCR results were assessed. The optimal concentration (20 μg/ml) of hUCMSCs secretion used in this study was determined by dose-dependent assay as shown in Fig. 1B. In time-dependent assay, the hBMSCs treated with hUCMSCs secretion factors showed more calcium deposits than did the OIM treated group in 3 days, 5 days and 7 days (Fig. 1C), implying that hUCMSCs secretion factors had promoted bone formation faster than the OIM. More important, calcium nodule formation was observed in the hUCMSCs secretion treated group rather than in the OIM group at day 3, suggesting that hUCMSCs secretion initiated osteogenic differentiation of MSCs by itself without OIM. The difference in mineralization between the hUCMSCs secretion group and OIM + hUCMSCs secretion group was not significant, suggesting that there is no synergistic effect between the hUCMSCs secretion and OIM on the osteogenesis of MSCs. Expression of osteogenic differentiation marker genes including ALP, BMP2, OCN, Osterix, Runx2 and Col1α were significantly up-regulated by the treatment of 20 μg/ml hUCMSCs secretion with or without OIM compared to control (Fig. 2). Although the difference in gene expression between the hUCMSCs secretion group and OIM + hUCMSCs secretion group was not obvious, the gene expression levels of both groups were significantly higher than those of the control group. Runx2, ALP, Col1 and BMP2 are responsible for osteoblast differentiation at early stage, while OCN is a marker gene at late stage [32]. ALP plays an essential role in producing bone mineral. It is a marker for cells that are undergoing osteoblast differentiation [33]. Runx2 is a key transcription factor that determines the maturation of osteoblasts [34]. As a downstream gene of Runx2, Osterix is an essential transcription factor for osteoblast differentiation and bone formation [35]. BMP2 plays a key regulatory role as a cell–cell signaling molecule in bone formation and repair [36]. The results implied that hUCMSCs secretion might be associated with the BMP2 signaling pathway, having the effect of enhancing osteogenic differentiation of MSCs. Therefore, hUCMSCs secretion factors may contain factors that promote the entire osteogenic differentiation process of MSCs. In addition, Col1α, a chondrogenic differentiation marker gene, was also significantly up-regulated by hUCMSCs secretion factors treatment compared to control. Previous study demonstrated that the cartilage-bone transition was a process of endochondral ossification in which cartilage was gradually replaced by bone under strict regulation of multiple signaling pathways [37]. Hence, the up-regulation of Col1α implied that the chondrogenic differentiation of hBMSCs may be augmented by hUCMSCs secretion factors too.

HA/TCP scaffold was widely used as bone substitute in orthopaedics research [38]. The results of ectopic bone formation in nude mice showed that more osteoid matrices formed in the BMSCs together with hUCMSCs secretion group than in either the BMSCs group or hUCMSCs secretion group. Because there is only limited blood supply in the dorsal skin of nude mice, either the hUCMSCs secretion or the BMSCs group did not induce sufficient bone formation respectively. However, the combination of hUCMSCs secretion factors and BMSCs had synergetic effects on promoting bone formation. Our result demonstrated that the treatment of BMSCs + hUCMSCs secretion was an improved solution for bone formation compared with using MSCs or hUCMSCs secretion factors alone.

Calvarial defect is a non-load bearing bone healing model, and one of the most commonly used defect models in bone reconstruction [39]. Critical size bone defects cannot heal without the assistance of therapeutic aids or materials designed to encourage bone regeneration [40]. Allogenic rBMSCs derived from GFP transgenic SD rat were used in the graft to trace the origin of the newly formed tissues. They also provided plenty of MSCs supplying the osteogenic differentiation process promoted by hUCMSCs secretion factors. Hyaluronic acid hydrogels have a significant impact on the differentiation of encapsulated MSCs [41]. No hyaluronic acid hydrogel was observed in the defect areas at sample collection, indicating that hyaluronic acid hydrogel was completely absorbed within 4 weeks. Safranin O staining showed a large amount of cartilage formation among the new bone tissues (Fig. 4D). Chondrocyte differentiation was the fundamental process in skeletal development. The majority of skeletal elements were formed through endochondral bone formation, which was characterized by the initial formation of cartilage molds from mesenchymal condensations and their subsequent replacement by bone [42]. OCN immunohistochemistry staining showed that the number of osteogenic cells was increased after treated by hUCMSCs secretion factors compared to control. The distribution of GFP-positive cells were consistent with OCN-positive cells, bone and cartilage in newly formed tissue, indicating that a certain amount of newly formed bone and cartilage tissue were originated from allogenic rBMSCs implanted in the defect instead of cells infiltrated from surrounding tissue. We observed some GFP positive cells in the form of clusters, surrounded in vesicle-like structures in cartilage, suggesting that some GFP cells trapped in the hyaluronic acid hydrogel had generated cartilage tissue.

Further, strict quality control must be implemented before hUCMSCs secretion factors are used in clinical applications. The limitations and risks are concerned, such as possible contamination, potential immune responses and presence of unknown viruses. Recombinant human bone morphogenetic protein-2 (rhBMP-2) is effective for osteogenic differentiation and it is applied to the treatment of orthopaedic diseases [43]. The ingredients of secretion factors are more complicated compared to the rhBMP-2. The various ingredients and safety issues of using hUCMSCs secretion factors need to be carefully defined in future studies.

## Supporting Information

S1 Fig Flow cytometery characterization of human fetal bone marrow derived mesenchymal stem cells. MSCs were collected and washed with PBS, then incubated with fluochrome-conjugated antibodies against CD34, CD44, CD45, CD73, CD90, CD105, and corresponding isotype control. The stained cells were immediately subjected to flow cytometric analysis using LSRFortessa Cell Analyzer. The cells were positive for CD44, CD73, CD90 and CD105 while negative for CD34 and CD45.
(DOCX)
