# Supplementary material for: The Effects of Secretion Factors from Umbilical Cord Derived Mesenchymal Stem Cells on Osteogenic Differentiation of Mesenchymal Stem Cells
Source: PLoS One. 2015 Mar 23;10(3):e0120593. doi: 10.1371/journal.pone.0120593 (PMC4370627; doi:10.1371/journal.pone.0120593)
Supplement: S1 Fig — MSCs were collected and washed with PBS, then incubated with fluochrome-conjugated antibodies against CD34, CD44, CD45, CD73, CD90, CD105, and corresponding isotype control. The stained cells were immediately subjected to flow cytometric analysis using LSRFortessa Cell Analyzer. The cells were positive for CD44, CD73, CD90 and CD105 while negative for CD34 and CD45. (DOCX) [file pone.0120593.s001.docx]

**
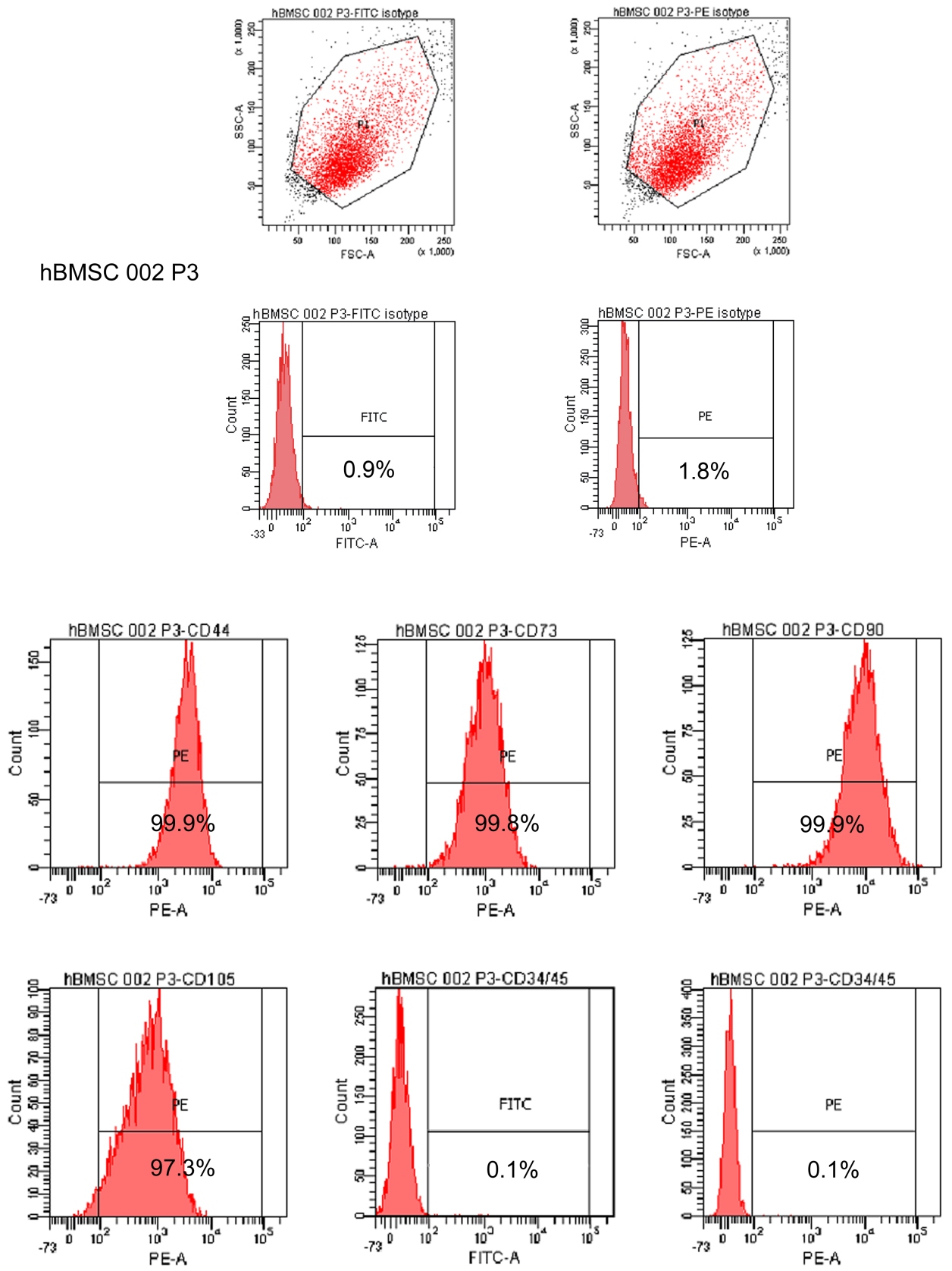
**

**Figure S1. Flow cytometery characterization of human fetal bone marrow derived mesenchymal stem cells.** MSCs were collected and washed with PBS, then incubated with fluochrome-conjugated antibodies against CD34, CD44, CD45, CD73, CD90, CD105, and corresponding isotype control. The stained cells were immediately subjected to flow cytometric analysis using LSRFortessa Cell Analyzer. The cells were positive for CD44, CD73, CD90 and CD105 while negative for CD34 and CD45.
